# Pavlovian bias in Parkinson’s disease: an objective marker of impulsivity that modulates with deep brain stimulation

**DOI:** 10.1038/s41598-020-69760-y

**Published:** 2020-08-10

**Authors:** Robert S. Eisinger, Bonnie M. Scott, Anh Le, Elena M. Torres Ponce, Joseph Lanese, Christopher Hundley, Brawn Nelson, Tasmeah Ravy, Janine Lopes, Sable Thompson, Sneha Sathish, Rebecca L. O’Connell, Michael S. Okun, Dawn Bowers, Aysegul Gunduz

**Affiliations:** 1 Department of Neuroscience, University of Florida, 1275 Center Drive, Gainesville, FL 32611 USA; 2 Department of Clinical and Health Psychology, University of Florida, Gainesville, FL USA; 3 Department of Neurology, Norman Fixel Institute for Neurological Diseases, University of Florida, Gainesville, FL USA; 4 J. Crayton Pruitt Department of Biomedical Engineering, University of Florida, Gainesville, FL USA

**Keywords:** Motivation, Decision, Reward, Parkinson's disease

## Abstract

Impulsivity is a common symptom in Parkinson’s disease (PD). Adaptive behavior is influenced by prepotent action-reward and inaction-avoid loss Pavlovian biases. We aimed to assess the hypothesis that impulsivity in PD is associated with Pavlovian bias, and to assess whether dopaminergic medications and deep brain stimulation (DBS) influence Pavlovian bias. A PD DBS cohort (N = 37) completed a reward-based Go/No-Go task and bias measures were calculated. This DBS cohort completed the task under three conditions: on-med/pre-DBS, off-med/off-DBS, and on-med/on-DBS. Participants also completed self-reported measures of impulsivity. Dopaminergic medication was associated with lower action-reward bias while DBS was associated with higher action-reward bias. Impulsivity was associated with higher action-reward bias but not inaction-avoid loss bias. We furthermore replicated this association in an independent, non-DBS PD cohort (N = 88). Overall we establish an objective behavioral marker of impulsivity and show that DBS affects impulsivity by amplifying automated responding. Our results point to the importance of reward rather than punishment avoidance in driving impulsive behaviors. This work provides insight into the pathophysiological underpinnings of impulsivity and especially medication and DBS-associated impulsivity in PD.

## Introduction

A fundamental, evolutionary purpose of the brain is to determine whether an organism should approach or avoid stimuli in the environment^1^. Failure during this process occurs in numerous neuropsychiatric diseases, including Parkinson’s disease (PD), and can manifest as one or more impulse control disorders (ICDs)—for example, pathological gambling, hypersexuality, binge eating, compulsive shopping, and hobbyism^2,3^. Among PD patients, ICDs are frequent and underdiagnosed^4,5^. ICDs and other non-motor symptoms in PD contribute more to poor quality of life than motor symptoms^6^. However, it is unclear why people with PD experience high rates of ICDs^2^. It is also unclear why effective PD treatments, including dopaminergic medications and deep brain stimulation (DBS) of either the subthalamic nucleus (STN) or globus pallidus internus (GPi), can exacerbate existing ICDs or precipitate the appearance of de novo ICDs^2,7–11^. Thus, there is a pressing need to understand the nature of impulsivity in PD, both in patients with and without DBS therapy.

Faced with a decision, impulsivity is associated with heightened reward sensitivity and deficient cognitive control, resulting in an ill-informed outcome to either act (“Go”) or inhibit action (“No Go”)^12,13^. Decades of work have investigated how such decisions materialize. For example, in 1986, researchers reported difficulty in training chickens to not approach a food reward in order to obtain it^14^. The automatic associations between approach and reward, and conversely, between avoidance and punishment, have been coined Pavlovian biases^3,15^. That is, an individual is more likely to act to obtain a reward than to avoid a punishment (action-reward bias); and an individual is more likely to *not* act to avoid a punishment than to obtain a reward (inaction-avoid loss bias)^16^. These anchoring biases implicate brain regions such as the striatum, ventral tegmental area, subthalamic nucleus, and frontal gyrus^17–22^.

Under normal circumstances, Pavlovian biases broadly influence adaptative behavior—they guide learning and form the basis for responding rapidly with little cognitive effort. However, they can become maladaptive^3,23–25^. People with PD in particular demonstrate difficulty in learning to withhold actions especially for reward outcomes^26,27^. ICDs in PD are specifically associated with impaired stopping behavior and response inhibition^10,28–30^, suggesting the possibility of an exaggerated action-reward Pavlovian bias. However, no study to date has assessed whether impulsivity in PD—especially in the context of dopaminergic medication and DBS therapy—is associated with abnormal Pavlovian biases. Such investigations in this vulnerable population are valuable because they could implicate pathological brain regions, establish objective behavioral correlates for clinical translation, and uncover mechanisms for the role of medications and DBS in affecting impulsivity.

In the present study, we used a reward-based, Pavlovian Go/No-Go paradigm to measure the action-reward bias (referred to simply as ‘reward bias’ throughout) and the inaction-avoid loss bias (referred to simply as ‘avoid-loss bias’ throughout) in PD patients with variable degrees of impulsivity measured using a standardized self-report questionnaire. We included a DBS cohort and a separate non-DBS cohort. In the DBS cohort, we further assessed the effect of medications and DBS on Pavlovian biases. Overall we expected performance to be higher during natural action-reward or inaction-avoid loss associations as compared to unnatural inaction-reward or action-avoid loss associations. Based on prior results from the Go/No-Go task in healthy adults and in patients with PD taking dopamine medications, we expected that the on-medication (on-med) state would be associated with decreased reward bias^16,26^. In addition, given that response inhibition is impaired in individuals with PD and ICD, we hypothesized that impulsivity would be associated with increased reward bias.

## Methods

### Participants

This is an Institutional Review Board (IRB)-approved study (IRB201601780 and IRB201702032) of two cohorts of adults with PD from the Movement Disorders Clinic at the University of Florida Norman Fixel Institute for Neurological Diseases. Written informed consent was obtained from all subjects in accordance with local guidelines and regulations. PD patients undergoing DBS at the University of Florida were recruited one day prior to their surgery. These individuals were deemed surgical candidates following an extensive multidisciplinary evaluation by neurology, neurosurgery, psychiatry, physical therapy, occupational therapy, neuropsychology, and speech and swallow therapy. DBS target (GPi or STN) and surgery type (unilateral or staged bilateral) were selected during this clinical evaluation as per standard of care. There was no randomization. We had no specific inclusion criteria for the DBS cohort beyond those used during this clinical evaluation. Exclusion criteria were severe vision or hearing impairment.

Participants in the non-DBS cohort were also recruited from the Movement Disorders Clinic. Inclusion criteria were right-hand dominant adults aged 50–80 with at least 8+ years of education, Beck Depression Inventory (BDI)-II less than 20, and Montreal Cognitive Assessment (MoCA) standardized score greater than 1.5 standard deviations below the mean. Exclusion criteria were current psychostimulant or cognitive-enhancing medication use, history of substance abuse, current unstable psychiatric disturbance (e.g., schizophrenia, bipolar disorder, etc.), other current neurological disturbance (e.g., traumatic brain injury, stroke, etc.), current chronic unstable medical conditions (e.g., metastatic cancer), history of brain surgery, and severe vision or hearing impairment.

### Study visits

The DBS cohort was enrolled in a study of intraoperative invasive electrophysiology during reward processing (Eisinger et al., Under Review). They played the Go/No-Go task one day before their DBS surgery in the on-med state. The majority of the participants (those that were not withdrawn) repeated the task the following day in the operating room during their DBS surgery in the off-med/off-DBS state (Table 1). Prior to surgery participants stopped dopaminergic medications for at least 12 h. We also attempted to follow participants postoperatively at clinic appointments in the on-med/on-DBS state. We aimed to gather multiple time points from the same patients in this state to study a possible learning effect of the task, which would be an important confounding factor (see Statistical Approach). Although some participants completed the task at multiple postoperative visits (see Table 1 footer), the task was only completed once at each visit. In the on-med/on-DBS condition, therapeutic DBS settings were used; thus, DBS settings were not adjusted for research purposes.

**Table 1:** Demographic and clinical characteristics of participants.

	DBS Cohort			Non-DBS Cohort	DBS vs non-DBS
	On-Med/Pre-DBS	Off-Med/Off-DBS	On-Med/On-DBS	On-Med	–
Timepoints (N)	37 (37)	34 (34)	47 (29)^a^	88 (88)	–
Age (M ± SD)	66.03 ± 9.30 yrs	66.68 ± 9.53 yrs	65.09 ± 10.01 yrs	67.36 ± 8.48 yrs	*P* = 0.51
Gender (F, M)	9, 28	8, 26	7, 40	31, 57	*P* < 0.05
Disease Duration (M ± SD)	10.13 ± 4.94 yrs	10.24 ± 5.09 yrs	10.38 ± 4.06 yrs	8.74 ± 6.41 yrs	*P* = 0.14
On-Med H&Y (M ± SD)	2.28 ± 0.08	–	2.21 ± 0.07	2.10 ± 0.08	*P* = 0.22
PDQ-39 Cognition (M ± SD)	23.44 ± 4.53	22.92 ± 4.75	22.67 ± 3.76	23.24 ± 2.10	*P* = 0.98
BDI-II (M ± SD)	9.14 ± 1.13	8.91 ± 1.21	8.64 ± 1.15	10.36 ± 0.99	*P* = 0.43
LEDD (M ± SD)	1,223 ± 810 mg	–	1,300 ± 1,172 mg	705 ± 594 mg	*P* < 0.001
DADD (M ± SD)	69.7 ± 102.9 mg	–	89.1 ± 106.0 mg	155.0 ± 78.8 mg	*P* < 0.05
QUIP-RS Total (M ± SD)	16.24 ± 10.05	15.59 ± 13.54	11.47 ± 11.49	15.65 ± 16.06	*P* = 0.60
Any ICD	21	19	16	34	*P* = 0.26
Gambling ICD	2	1	2	3	–
Sex ICD	4	4	2	7	–
Shopping ICD	1	1	2	5	–
Eating ICD	5	6	7	11	–
Hobbyism-Punding ICD	19	16	8	28	–
Medications ICD	2	1	1	5	–

M = mean, SD = standard deviation, F = female, M = male, LEDD = levodopa equivalency daily dose, DADD = dopamine agonist equivalency daily dose, QUIP-RS = questionnaire for impulsive compulsive disorders in Parkinson’s disease-rating scale, Yrs = years, mg = milligrams, ICD = impulse control disorder, PDQ-39 = Parkinson’s disease questionnaire, BDI-II = Beck Depression Inventory, H&Y = Hoehn & Yahr, DBS = deep brain stimulation, Med = medication.

^a^One participant completed it five times, two participants completed it three times, ten participants completed it two times, and the rest completed it once; 22 (14) bilateral GPi, 8 (6) unilateral GPi, 10 (5) bilateral STN, 7 (4) unilateral STN.

The non-DBS cohort was enrolled in a separate but related study of non-invasive electrophysiology during reward processing. They completed the Go/No-Go task at a single visit in the on-med state while wearing an electroencephalography cap. The data presented in this paper include the Go/No-Go behavioral results from these two cohorts.

### Behavioral task

The Go/No-Go paradigm consisted of trials that included stimulus presentation, action selection, and feedback presentation (Supplementary Figure 1a). The stimulus was a large square centered within the monitor that was colored blue (Go-To-Win [GTW]), yellow (Go-To-Avoid-Loss [GTAL]), orange (No-Go-To-Win [NGTW]), or pink (No-Go-To-Avoid-Loss [NGTAL]) (Supplementary Figure 1b). Participants had to either press a button (Go trials) or not (No-Go trials) to either win points or avoid losing points. Win trials performed correctly led to + 100 points and avoid-loss trials performed correctly led to + 0 points. Win trials performed incorrectly led to + 0 points and avoid-loss trials performed incorrectly led to − 100 points. Participants had 1000 ms to press the button otherwise the game would proceed (Supplementary Figure 1a). In the DBS cohort, a short 250 ms delay then led to feedback presentation for 500 ms. Feedback appeared in a gray rectangle on top of the stimulus in the center of the screen. In the DBS cohort there was a blank screen for 250 ms before the next trial began. In the non-DBS cohort each trial was preceded by a 1000 ms screen with the text “Ready”. Additionally, in the non-DBS cohort, a 2000 ms screen with a plus sign at the center preceded feedback, which was followed by a 2000 ms delay before the next trial. The slower pace of the task in the non-DBS cohort allowed us to test specific hypotheses about cortical event-related potentials during reward processing as part of a separate analysis (not shown). The DBS cohort completed 120 trials during each run whereas the non-DBS cohort completed 100 trials due to the slower design. For all visits stimuli were chosen randomly but were counterbalanced. Participants were instructed to try to earn as many points as possible while being as fast as possible, and were given the opportunity to practice trials until they felt that they understood the game. Our intent was not to study the learning phase of this task (see “Discussion” section).

### Experimental setup

Participants in the non-DBS cohort as well as in the on-med/pre-DBS and on-med/on-DBS condition of the DBS cohort completed the task seated in front of a desk using a laptop computer. During the off-med/off-DBS condition participants played the game while on the operating room table shortly after the DBS lead was implanted. An adjustable monitor attached to a rolling cart was positioned in front of the patient with an appropriate viewing angle. We cycled through the four different stimuli several times to ensure that participants could clearly view the game. Participants in the DBS cohort played the game with the hand contralateral to the implanted hemisphere. The button was a custom-made pressure-based sensor that triggered a button press when approximately 300 mg of force was applied. Participants held the button handle with their hand/fingers and used their thumb to press on the button which was fastened to the top of the handle. The button was connected to a custom-made circuit which triggered the button presses with Arduino software^31^. Participants in the non-DBS cohort played the game with their right hand and responded using the space bar on a keyboard.

### Demographic and clinical data

To characterize the cohorts we recorded age, gender, disease duration, the Beck Depression Inventory (BDI)-II, and the PD quality of life questionnaire (PDQ-39) cognition subscore for all participants. We additionally recorded the on-med Hoehn & Yahr at the on-med timepoints. We included BDI because it is known that impulsivity correlates to depression, and we used the PDQ-39 cognition subscore as a surrogate marker of cognitive ability. For the non-DBS cohort and for the on-med conditions of the DBS cohort we quantified dopaminergic medication use with a levodopa equivalency daily dose (LEDD), which included dopamine agonists, as well as the equivalency dose when only considering dopamine agonists (DADD). The Questionnaire for Impulsive-Compulsive Disorders in Parkinson’s Disease-Rating Scale (QUIP-RS)^32^ was completed by participants in person during study visits if time permitted or over the telephone if not. In the DBS cohort, the QUIP-RS, BDI-II, and PDQ-39 cognition scores from the on-med/pre-DBS visit were assumed to be unchanged the next day during the off-med/off-DBS visit. We categorized each of the ICDs in the QUIP-RS assessment—gambling, sex, shopping, eating, hobbyism-punding, and medication use—as present (positive [ICD+]) or absent (negative [ICD−]) according to established cutoffs^32^.

### Behavioral measures

All data analyses were completed in R 3.5.2 (r-project.org). Accuracy was computed as the proportion of trials correct and error was defined as a proportion of trials incorrect. Reaction time (RT) was computed as the time elapsed between stimulus presentation and button press on correct Go trials. Pavlovian bias was defined as the error rate of the unnatural valence-action association divided by the accuracy of the respective natural valence-action association. Namely, reward bias was defined as NGTW error divided by GTW accuracy, and avoid-loss bias was defined as GTAL error divided by NGTAL accuracy. This calculation thus represents a functional measure of a prepotent, impulsive action-reward association incorrectly applied to NGTW trials, and of a prepotent, impulsive inaction-avoid loss association incorrectly applied to GTAL trials. The numerator represents a raw bias and the denominator scales the bias by the strength of the natural association. For instance, a bias of 0 suggests that individuals were able to completely overcome prepotent associations—that is, throughout the entire task bias did not affect performance on unnatural associations. Hence, the presence of a Pavlovian bias occurring at the group level is indicated by a distribution of bias values greater than 0 (i.e., one-way statistical comparison). In contrast, a bias value greater than 1 suggests that the natural, prepotent association was weaker than the unnatural association. This would be highly unusual as it goes against the Pavlovian bias phenomenon and probably suggests that instructions were not properly followed during the task. We therefore removed these instances as outliers from the study (zero instances in the on-med/pre-DBS condition; two instances in the off-med/off-DBS condition; one instance in the on-med/on-DBS condition; one instance in the on-med, non-DBS cohort). The N listed in Table 1 represent the data after these outliers were removed.

### Statistical approach

To characterize the participants we first compared baseline characteristics between all visits of the DBS and non-DBS cohort using mixed model analyses of variance for continuous data and chi-square tests for categorical data. Mixed model analyses were used throughout this study to provide a method for controlling for possible confounding variables and for the random effect of participants, particularly because there were data at multiple visits from the same participants in the DBS cohort.

To analyze behavioral performance, we began by examining accuracy. We used a mixed model to test whether overall accuracy depended on the cohort (DBS or non-DBS). Further analyses were then carried out as necessary to interpret significant effects from mixed models, which led us to further split analyses by cohort and by condition within the DBS cohort. For instance, within the DBS cohort, we also tested for an effect of the med/DBS condition on overall accuracy. In addition to total accuracy, we also evaluated whether Go/No-Go accuracy depended on the trial condition. To do so we included terms in a mixed model for the cohort, trial valence (reward or avoid-loss), trial action (Go or No-Go), and all their interactions. This has been done in prior studies and led us to hypothesize higher accuracies in GTW vs NGTW and in NGTAL vs GTAL in each of these one-tailed comparisons, respectively. Similarly, a predicted result of Pavlovian bias is that RT should be faster in GTW versus GTAL trials, and we therefore tested this in our data with one-tailed comparisons as well.

Given the possible influence of learning on Go/No-Go performance over repeated completions of the task in the on-med/on-DBS condition, we also included a variable in the mixed models for an effect of time since the first visit. In our models we also included terms for brain target (GPi or STN) and surgery type (unilateral or bilateral) as fixed effects for analyses within the on-med/on-DBS condition.

The primary measures of interest in this study were the Pavlovian biases. We evaluated reward and avoid-loss biases similar to accuracies—first checking for an effect of cohort, then for an effect of med/DBS condition within the DBS cohort, and finally for effects of brain target or stimulation type in the on-med/on-DBS cohort. This also led us to split reward and avoid-loss bias analyses by cohort and condition within the DBS cohort and to compare reward but not avoid-loss bias across different conditions within the DBS cohort (see “Results” section). To evaluate the hypothesis that Pavlovian biases were present in all cohorts and conditions, we used one-way Wilcoxon signed-rank tests for reward and avoid-loss biases because these are non-normal distributions due to the lower limit of 0. We then determined whether reward and avoid-loss biases depended on QUIP-RS using mixed models that simultaneously controlled for age, LEDD, disease duration, gender, PDQ-39 cognition, and BDI-II scores. In previous studies these factors have been found to relate to QUIP-RS^2^. In a related analysis, we compared both reward and avoid-loss biases across participants categorized as ICD-negative and ICD-positive.

Throughout our analyses specific comparisons across conditions or cohorts were completed by student t-tests or Wilcoxon signed-rank tests for normal or non-normal data, respectively. Normality was assessed using Shapiro–Wilk tests. Lastly, we use Pearson or Spearman correlations for normal or non-normal data, respectively, to evaluate the relationship between impulsivity and Pavlovian biases as well as other clinical factors.

## Results

### Sample characteristics

A total of 37 people with PD in the DBS cohort completed the Go/No-Go task (Table 1). Each participant completed the task once in the on-med/pre-DBS state and 34 of these participants completed the task one day later in the off-med/off-DBS state. 29 participants from the DBS cohort also completed the task in the on-med/on-DBS state a total of 47 times (30 GPi, 17 STN), separated by 121 ± 70 days (Mean ± SD, range, 34–287) between sessions. The complete breakdown for the number of participants with unilateral or bilateral and GPi or STN stimulation is provided in Table 1. A separate non-DBS cohort of 88 people with PD also completed the task in the on-med state.

DBS and non-DBS cohorts were similar with regards to age and disease duration at the time of task completion (Table 1). The PDQ-39 cognition subscore, BDI scores, and Hoehn & Yahr scores were also similar. The non-DBS cohort included more female participants than the DBS cohort (X_1_^2^ = 4.97, *P* < 0.05). Participants in the DBS cohort tended to use more dopaminergic medications (F_1,117_ = 15.42, *P* < 0.001) despite showing similar QUIP-RS scores and similar proportions of participants with any ICD(s) (Table 1).

### Accuracy and reaction time

Overall accuracy across all trials during the Go/No-Go task was significantly lower in the DBS cohort compared to the non-DBS cohort (0.90 ± 0.01 vs 0.96 ± 0.01, F_1,139_ = 27.74, *P* < 0.001). Within the DBS cohort, there was no effect of med/DBS condition on overall accuracy across all trials (*P* = 0.29). In addition, within the on-med/on-DBS condition of the DBS cohort, overall accuracy did not depend on brain target (GPi, 0.88 ± 0.02; STN, 0.94 ± 0.01; GPi vs STN, *P* = 0.069), surgery type (unilateral vs bilateral surgery) (*P* = 0.82), time since the first visit (*P* = 0.82), or any of their interactions.

**Figure 1:**
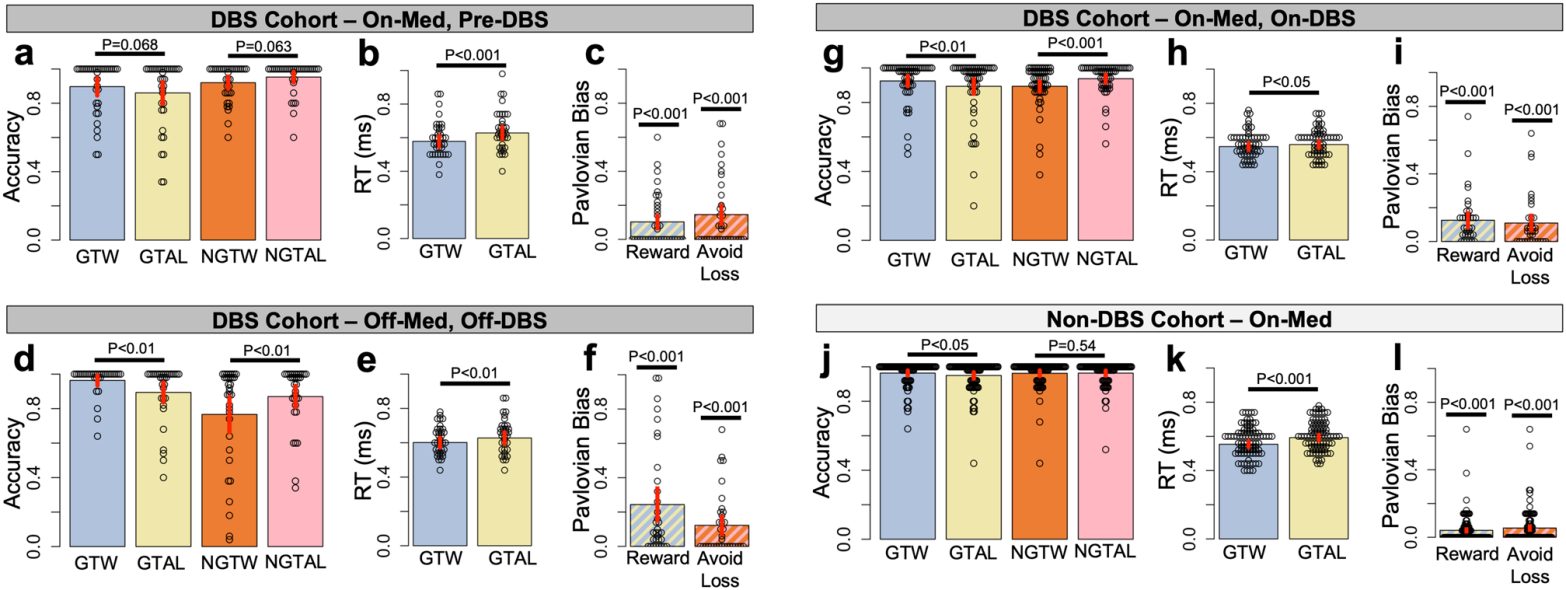
Performance and pavlovian bias during the Go/No-Go task. Dark gray headers refer to the DBS cohort (**a**–**i**) and the light gray header refers to the non-DBS cohort (**j**–**l**). The DBS cohort is split across three med/DBS conditions: on-med/pre-DBS (**a**–**c**); off-med/off-DBS (**d**–**f**); on-med/on-DBS (**g**–**i**). Each panel includes accuracy (**a**,**d**,**g**,**j**), reaction time (RT) (**b**,**e**,**h**,**k**), and Pavlovian biases (**c**,**f**,**i**,**l**). Average accuracy during each trial condition is shown in blue for Go-To-Win (GTW), yellow for Go-To-Avoid-Loss (GTAL), orange for No-Go-To-Win (NGTW) and pink for No-Go-To-Avoid-Loss (NGTAL) (colors correspond to Supplementary Figure 1). RT is shown in blue for GTW and in yellow for GTAL. Pavlovian biases are shown for reward bias in striped blue-yellow and for avoid-loss bias in striped orange-pink. Red bars in each barplot indicate two standard errors above and below the mean. *P*-values above bars for accuracy and RT indicate results of one-tailed comparisons and *P*-values above bars for Pavlovian biases indicate one-way comparisons (see “Methods” section).

On the other hand, accuracies during different trial conditions depended on cohort (DBS vs non-DBS), trial valence (reward vs avoid-loss), and trial action (Go vs No-Go) through a cohort main effect (F_1,135_ = 31.17, *P* < 0.001), a valence by action interaction (F_1,726_ = 11.07, *P* < 0.001), and a three-way valence by action by cohort interaction (F_1,726_ = 6.92, *P* < 0.01). We therefore separated this analysis for each cohort. Within the DBS cohort, accuracy depended on a valence by action interaction (F_1,421_ = 15.16, *P* < 0.001), and an action by med/DBS condition interaction (F_2,421_ = 11.75, *P* < 0.001). Thus for the DBS cohort we also separated accuracy data across the different med/DBS conditions. Within the on-med/on-DBS group, accuracy depended on an interaction between action and valence (F_1,146_ = 6.08, *P* < 0.05) but not on any other main effects or interactions including brain target or surgery type. Considering these effects overall, we compared accuracy across GTW, GTAL, NGTW, and NGTAL trials and across cohorts, as well as across med/DBS conditions within the DBS cohort, but not across brain targets or surgery type in the on-DBS condition (Fig. 1).

Performance in the DBS cohort overall reflected prepotent Pavlovian behavior with higher GTW versus GTAL accuracy (0.93 ± 0.01 vs 0.88 ± 0.02, V = 2,211, *P* < 0.001) as well as lower NGTW versus NGTAL accuracy (0.86 ± 0.02 vs 0.92 ± 0.01, V = 994, *P* < 0.001). This effect was further seen specifically in the off-med/off-DBS condition (Fig. 1d) and the on-med/on-DBS condition (Fig. 1g). However, in the on-med/pre-DBS condition these relationships only exhibited a statistical trend (Fig. 1a). In the non-DBS cohort accuracies were especially high with a notable ceiling effect (Fig. 1j). Only GTW accuracy was significantly higher than GTAL accuracy (0.96 ± 0.01 vs 0.95 ± 0.01, V = 521, *P* < 0.05).

Another predicted result of the Pavlovian bias in this task is that RT should be faster during GTW compared to GTAL trials. This effect was observed in the on-med/pre-DBS condition (Fig. 1b), the off-med/off-DBS condition (Fig. 1e), and in the on-med, non-DBS cohort (Fig. 1k). This effect exhibited a statistical trend in the on-med/on-DBS condition (Fig. 1h). In all cases effect sizes were quite small (e.g., for the on-med/pre-DBS condition, 0.58 ± 0.02 vs 0.63 ± 0.02 s, t_36_ =  − 3.66, *P* < 0.001). Taken together, accuracy and RT results suggest that participants performed the task as expected, but they likely prioritized accuracy over speed. Additionally, the task was clearly less challenging for the non-DBS cohort.

### Action-reward bias and inaction-avoid loss bias

Next we computed reward and avoid-loss biases during the Go/No-Go task. Similar to accuracies across Go/No-Go conditions, both reward (F_1,146_ = 20.86, *P* < 0.001) and avoid-loss biases (F_1,138_ = 12.32, *P* < 0.01) differed between the DBS and non-DBS cohorts (reward bias, 0.16 ± 0.02 vs 0.04 ± 0.01, W = 7,138, *P* < 0.001; avoid-loss bias, 0.13 ± 0.02 vs 0.06 ± 0.01, W = 6,351, *P* < 0.01). Reward but not avoid-loss bias further depended on the med/DBS condition within the DBS cohort (reward bias, F_2,89_ = 5.17, *P* < 0.01; avoid-loss bias, *P* = 0.74). Reward and avoid-loss bias within the on-med/on-DBS condition did not depend on brain target or stimulation type. Given these effects we also separated biases by the different cohorts and conditions within the DBS cohort (Fig. 1). Biases were significantly greater than zero in the on-med/pre-DBS condition (reward bias, *P* < 0.001; avoid-loss bias, *P* < 0.001; Fig. 1a), in the off-med/off-DBS condition (reward bias, V = 351, *P* < 0.001; avoid-loss bias, V = 153, *P* < 0.001; Fig. 1b), in the on-med/on-DBS condition (reward bias, V = 703, *P* < 0.001; avoid-loss bias, V = 528, *P* < 0.001; Fig. 1c), and in the on-med, non-DBS cohort (reward bias, V = 666, *P* < 0.001; avoid-loss bias, V = 741, *P* < 0.001; Fig. 1d). Bias magnitudes across cohorts were significantly associated with overall mean accuracy (reward bias, rho =  − 0.70, *P* < 0.001; avoid-loss bias, rho =  − 0.73, *P* < 0.001). Similarly, across all participants and conditions, higher reward biases were significantly associated with higher avoid-loss biases (rho = 0.26, *P* < 0.001).

### Relationship between Pavlovian bias and impulsivity

Our primary investigation was whether Pavlovian biases depended on impulsivity. To explore possible confounding variables, we first examined the relationship between impulsivity and other clinical factors that have previously been associated with impulsivity (Supplementary Figure 2). QUIP-RS was not associated with age (Supplementary Figure 2a,b), gender (Supplementary Figure 2e), disease duration (Supplementary Figure 2f,g), or LEDD or DADD (Supplementary Figure 2h,i). However, we found positive associations between impulsivity and PDQ-39 cognition (Supplementary Figure 2c,d), and between impulsivity and BDI (Supplementary Figure 2j,k).

In mixed model analyses, reward bias depended only on QUIP-RS (F_1,138_ = 7.05, *P* < 0.01) and the cohort (F_1,116_ = 16.53, *P* < 0.001)—whereas avoid-loss bias only depended on the cohort (F_1,104_ = 7.92, *P* < 0.01) and BDI (F_1,141_ = 4.11, *P* < 0.05). Both reward and avoid-loss biases did not depend on age, levodopa dose, disease duration, gender, or PDQ-39 cognition subscore. Reward bias was slightly lower in the on-med/pre-DBS condition compared to the on-med/on-DBS condition (0.10 ± 0.03 vs 0.13 ± 0.02, W = 675, *P* < 0.05; Fig. 1c,i). Reward bias was much lower in the on-med/pre-DBS condition compared to the off-med/off-DBS condition (0.10 ± 0.03 vs 0.24 ± 0.05, W = 436, *P* < 0.05; Fig. 1c,f). Reward bias was not significantly different in the off-med/off-DBS condition compared to the on-med/on-DBS condition (*P* = 0.48; Fig. 1f,i).

**Figure 2:**
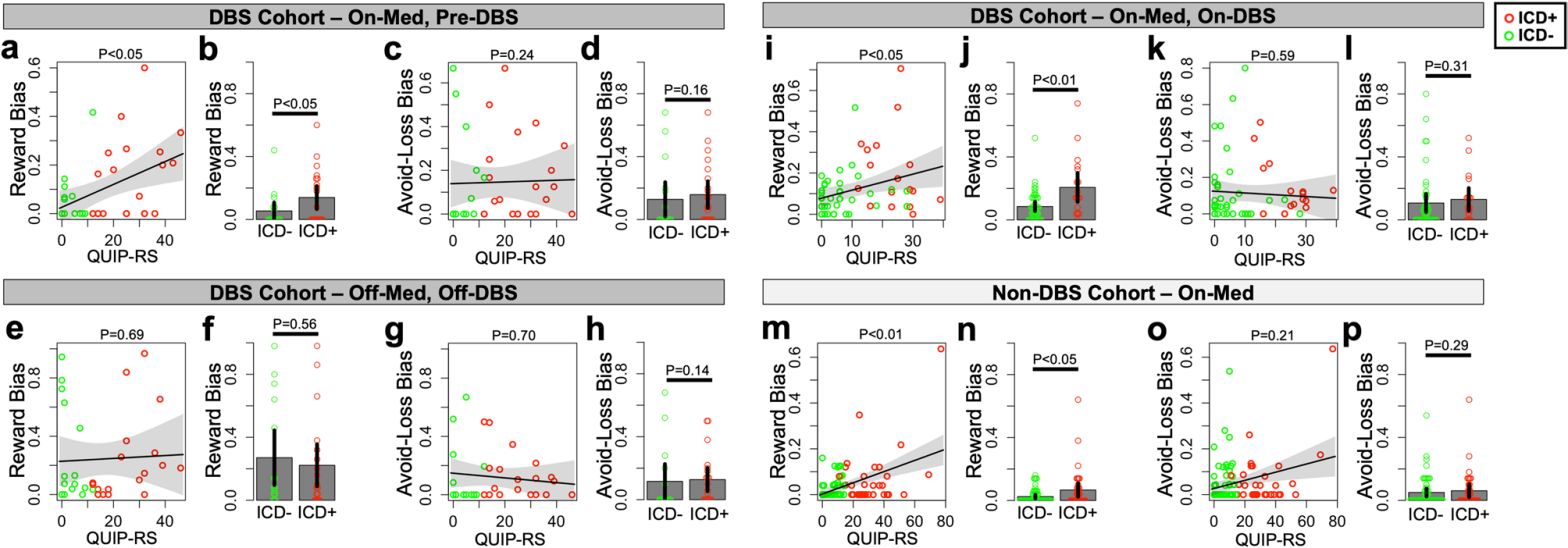
Relationship between impulsivity and pavlovian bias during the Go/No-Go task. Dark gray headers refer to the DBS cohort (**a**–**h**) and the light gray header refers to the non-DBS cohort (**m**–**p**). The DBS cohort is split across three med/DBS conditions: on-med/pre-DBS (**a**–**d**); off-med/off-DBS (**e**–**h**); on-med/on-DBS (**i**–**l**). Each panel includes a correlation between QUIP-RS and reward bias (**a**,**e**,**i**,**m**) as well as a comparison of reward bias among ICD-negative (ICD-) and ICD-positive (ICD+) participants (**b**,**f**,**j**,**n**). Each panel also includes a correlation between QUIP-RS and avoid-loss bias (IALB) (c,g,k,o) as well as a comparison of avoid-loss bias among ICD- and ICD+ participants (**d**,**h**,**l**,**p**). *P*-values above the correlations indicate the significance of the association between QUIP-RS and the bias shown. *P*-values above the barplots indicate significance of the two-tailed comparison between ICD- and ICD+ patients. Throughout, green dots indicate data points from ICD- participants and red dots indicate data points from ICD+ participants. Note that with the datapoint at QUIP-RS = 80 in (**m**) removed, the correlation remains significant at *P* < 0.05.

We next examined relationships between QUIP-RS and reward or avoid-loss biases across cohorts and within the different med/DBS conditions of the DBS cohort (Fig. 2). In the DBS cohort, QUIP-RS was associated with reward bias in the on-med/pre-DBS condition (rho = 0.40, *P* < 0.05; Fig. 2a) and in the on-med/on-DBS condition (rho = 0.31, *P* < 0.05; Fig. 2i), but not in the off-med/off-DBS condition (*P* = 0.69; Fig. 2e). QUIP-RS was also significantly associated with reward bias in the on-med/non-DBS cohort (rho = 0.30, *P* < 0.01; Fig. 2m). These relationships are also reflected in comparisons of reward biases between participants considered ICD-negative and ICD-positive (Fig. 2b,f,j,n). Avoid-loss biases were not associated with QUIP-RS in any cohort or condition (Fig. 2c,d,g,h,k,l,o,p).

## Discussion

To our knowledge this is the first study to investigate the relationship between impulsivity and Pavlovian bias in PD patients. Our study also assessed the influence of medications and DBS on these relationships. Overall in two independent cohorts we found that impulsivity in PD is associated with higher reward bias. Therapies for PD were also associated with changes in reward bias.

It is important to note that a key distinction between many previous studies and the present investigation is that in our Go/No-Go paradigm, participants were explicitly told what the stimulus-action-outcome associations were rather than learning them through trial and error during the task^16,18,19,26,33^. This can be interpreted within the context of real-life situations relevant for ICDs in which impulsivity can persist far beyond the learning phase. For instance, impulsive individuals with punding are likely not constantly re-learning how to sort objects or tidy up around the house. In addition, our data revealed a lack of a learning effect in the on-med/on-DBS cohort in which some individuals completed the task more than once at separate timepoints. Taken together, we consider performance in this study to represent previously learned associations.

Performance during this task reflected typical performance for similar Pavlovian Go/No-Go tasks completed by healthy populations^16,17,19,20,24,33,34^. That is, participants overall showed an action-reward association and an inaction-avoid loss association. Similarly, reaction time was generally faster when acting for reward compared to acting to avoid loss. It was not surprising that the non-DBS cohort failed to show a difference in performance between NGTW and NGTAL trials. We were anticipating a possible ceiling effect particularly in this more moderately-diseased non-DBS PD cohort, which has also been seen in healthy adults^20^. The overall speed of the task was slower for the non-DBS participants (see “Methods” section) and thus was more likely perceived as easier. Pavlovian biases would probably emerge more robustly with a faster paced task with more limited decision time. In addition, these relationships were only marginal in the on-med/pre-DBS state. Nonetheless, in all groups we were able to detect significantly elevated reward and avoid-loss biases, suggesting the presence of Pavlovian biases.

In this study reward biases were lower in the on-med setting. This finding was consistent with a study of healthy individuals given levodopa prior to completing a similar Go/No-Go task^16^. The researchers found that levodopa resulted in higher NGTW accuracy compared to placebo. This effect was also recently replicated in a PD cohort^26^. If ICD is associated with higher reward bias in the on-med state, and if dopaminergic medications are associated with reduced reward bias, then this adds to a growing body of literature challenging the prevailing hypothesis that ICDs in PD result from dopamine use^2^. Indeed, we did not see a dose-dependent relationship between QUIP-RS and levodopa equivalency dose or dopamine agonist dose in this sample, consistent with other reports^35–39^. Similarly, the DBS cohort at baseline showed greater dopamine use than the non-DBS cohort despite similar rates of ICD. However, it is important to note that dopamine could be influencing ICDs via other mechanisms. Dopamine affects motivation through reward-guided learning as well as through its role in movement vigor and other higher-order cognitive functions^18^. In this study, the difference in bias between the on-med/pre-DBS state and the on-med/on-DBS state is unlikely to have been due to movement vigor, as RTs were quite similar. In addition, prior work with a non-rewarding Go/No-Go task showed that dopamine in fact can reduce motor impulsivity^40^. We did not evaluate the learning process during this task; this and other cognitive functions reliant on dopamine in the context of PD ICDs remain open to investigation.

In contrast to the association of medications with decreased reward biases, we found that the on-DBS state resulted in higher reward biases relative to the on-med/pre-DBS state. In addition to being in the off-med state, it is also possible that a temporary lesion effect following DBS implantation contributed to higher reward biases seen in the off-med/off-DBS setting, and this will require further studies to carefully tease apart. Pavlovian biases in the on-DBS state did not depend on whether the stimulation was in the GPi or STN. It is known that DBS is a risk factor for development of ICDs^11,41–49^, and it is conceivable that this could be related to prepotent Pavlovian responding^50–54^. Very little is known about the role of the human GPi in impulsivity. Elevated reward biases in the context of GPi DBS could be related to altered habit formation or valence encoding^55,56^. On the other hand, STN stimulation is thought to cause impulsive responding by lowering the decision threshold for actions that are based on accumulated evidence^54,57–65^. The STN plays a critical role in stopping behavior within the cortico-striato-thalamo-cortical circuit, particularly via the hyperdirect pathway and the prefrontal cortex^50,54,66,67^. Fast-acting Pavlovian responses thereby act in opposition to the STN’s braking mechanism for decision-making. In line with our results, this would be characterized by higher Pavlovian biases in the on-DBS condition. Furthermore, this was only the case for reward and not avoid-loss bias, suggesting that DBS could specifically amplify automated action responding in reward contexts. Though in this study we only considered stimulation to therapeutic DBS contacts, it has been suggested that stimulation-induced prepotent responding occurs specifically with ventral (limbic) STN stimulation^53^. The isolated effects of DBS on Pavlovian biases should be ascertained in future work with a postoperative DBS cohort in the off-med/on-DBS setting.

An important observation from this study was that impulsivity was associated with increased Pavlovian bias, and in particular, action-reward association bias. This effect was present independent of age, LEDD, disease duration, gender, PDQ-39 cognition, and BDI-II. Interestingly, across all participants, higher reward biases were also associated with higher avoid-loss biases—however, QUIP-RS was only correlated to reward biases. The only condition for which this was not the case was the off-med/off-DBS state. This is an atypical state for PD patients which does not usually occur in circumstances outside of clinical assessment. The QUIP-RS assessment used at this time point captures individuals in their day-to-day activities (on-med) and so in hindsight it was unsurprising that this relationship did not hold. Despite a notable ceiling effect of accuracy in the non-DBS group, which led to low action-reward biases, the variability in those bias values were still related to QUIP-RS. This is an encouraging finding which points to the potential sensitivity of this task in deriving an objective marker of impulsivity even in PD populations that perform particularly well.

Overall, throughout the different groups and conditions in this study, the fact that reward but not avoid-loss bias was higher in people with ICD points to the importance of reward, and not loss avoidance, in driving impulsive behaviors. This finding also suggests that ICDs in PD are not simply a motor or a general executive dysfunction phenomenon, in which we would expect both reward and avoid-loss biases to be equally impacted. This distinction can only be appreciated in tasks such as the one used here that orthogonalizes positive and negative valence with action and inaction conditions. Whereas punishment drives faster motor learning, reward more so enhances retention of learned motor sequences^68^. Therefore, hypersensitivity to reward anticipation and reward feedback, which drive impulsive choices, probably underpin the persistence of ICDs.

This work contributes to a growing literature on Pavlovian biases in neuropsychiatric diseases. For example, people with schizophrenia exhibit reinforcement learning deficits with an associated reduction in the Go bias^23^. On the other hand, traumatic stress is associated with increased Pavlovian biases^24^ while anxiety is specifically associated with increased avoidance bias in the face of threats^25^. People with depression show similar Pavlovian bias to healthy controls^69^, though interestingly in this study we found an association between avoid-loss bias and BDI. The functional meaning of this association is unclear and will require further studies, though we suspect it could point to an abnormal loss sensitivity in depression. Given that the Pavlovian bias fundamentally influences basic human behavior, it is likely that Pavlovian dysfunction will be assessed in future work across many more diseases.

This study has important limitations. First, the off-med/off-DBS condition of the DBS cohort was carried out in an operating room setting whereas the other conditions involved participants sitting comfortably at a desk. The operating room environment can induce discomfort, which could affect performance during the task. Second, the on-DBS condition involved different numbers of visits across participants. However, this was intentional to study a possible learning effect, and we included a random effect term in our mixed model analyses to control for this methodological consideration. Third, stimulation configuration in the on-DBS condition followed from therapeutic settings and we did not systematically stimulate specific DBS contacts. However, this improves the generalizability of our results to PD DBS patients (e.g., as opposed to patients specifically receiving bilateral, ventral STN stimulation). Fourth, we did not specifically recruit balanced numbers of unilateral and bilateral GPi and STN participants, and the study may not have been sufficiently powered to fully appreciate effects of these variables. For example, the GPi vs STN effect of overall mean accuracy was trending, but did not reach statistical significance. Similarly, we also did not specifically recruit patients with certain PD ICDs, and it is possible that the Pavlovian biases relate to some ICDs more than others. Fifth, the Go/No-Go task in the non-DBS cohort was slower paced than the task used in the DBS cohort, though participants still had 1000 ms to respond to the stimuli. The slower paced design probably contributed to higher accuracies. Nonetheless, we were able to replicate the relationship between QUIP-RS and reward bias seen in the DBS cohort. Lastly, the different conditions of the DBS cohort in this study were all completed in the same sequence: on-med/pre-DBS, then off-med/off-DBS, then on-med/on-DBS. Future work could include an off-med/off-DBS condition postoperatively, as well as an off-med/on-DBS condition.

In conclusion, this is the first study to establish Pavlovian bias abnormalities in PD patients with ICDs. We demonstrate that simple and objective behavioral measures during a Go/No-Go task in part captures subjective impulsivity scores in people with PD. We found that Pavlovian biases are affected by therapies used in PD including dopaminergic medication and DBS of either the STN or GPi. Finally, total impulsivity severity was associated with increased reward bias, but not avoid-loss bias, hence pointing to the importance of reward-guided behaviors for ICDs. This finding was replicated in a separate cohort of PD patients. Future studies should further clarify why these deficits occur in people with PD.

## Supplementary information

Supplementary file 1.

Supplementary file 2.

## Data Availability

The datasets analyzed during the current study are available from the corresponding author on reasonable request.
