# Supplementary figures and images for: Pavlovian bias in Parkinson’s disease: an objective marker of impulsivity that modulates with deep brain stimulation
Source: Sci Rep. 2020 Aug 10;10:13448. doi: 10.1038/s41598-020-69760-y (PMC7417529; doi:10.1038/s41598-020-69760-y)

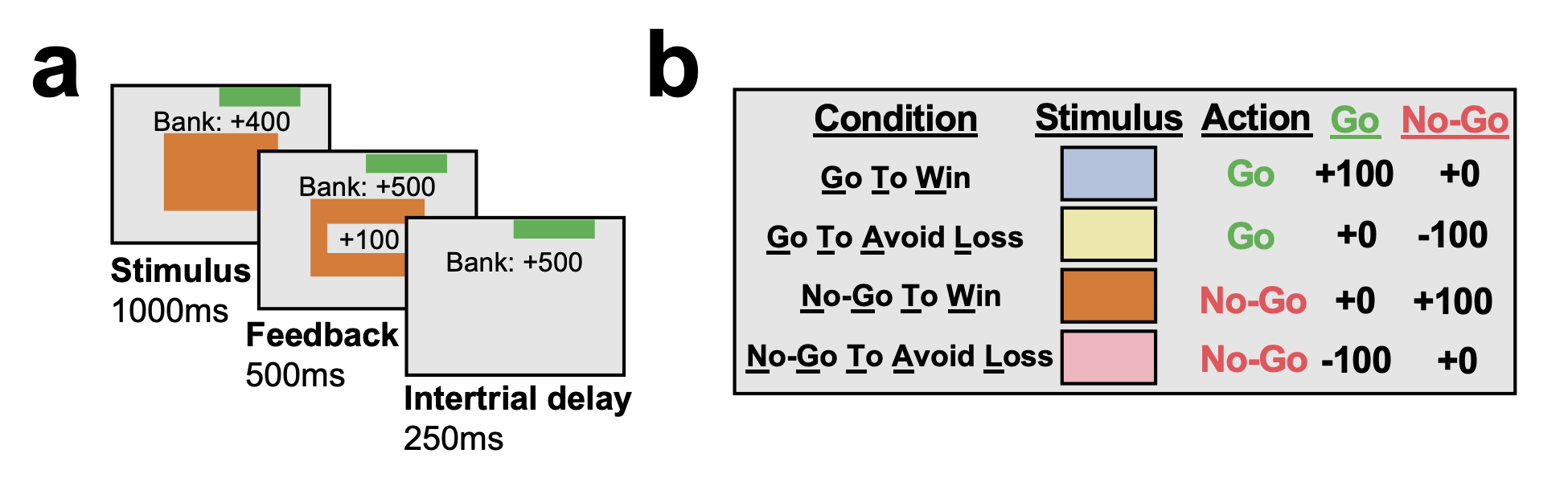

Supplement: Supplementary file 1 [file 41598_2020_69760_MOESM1_ESM.tiff]

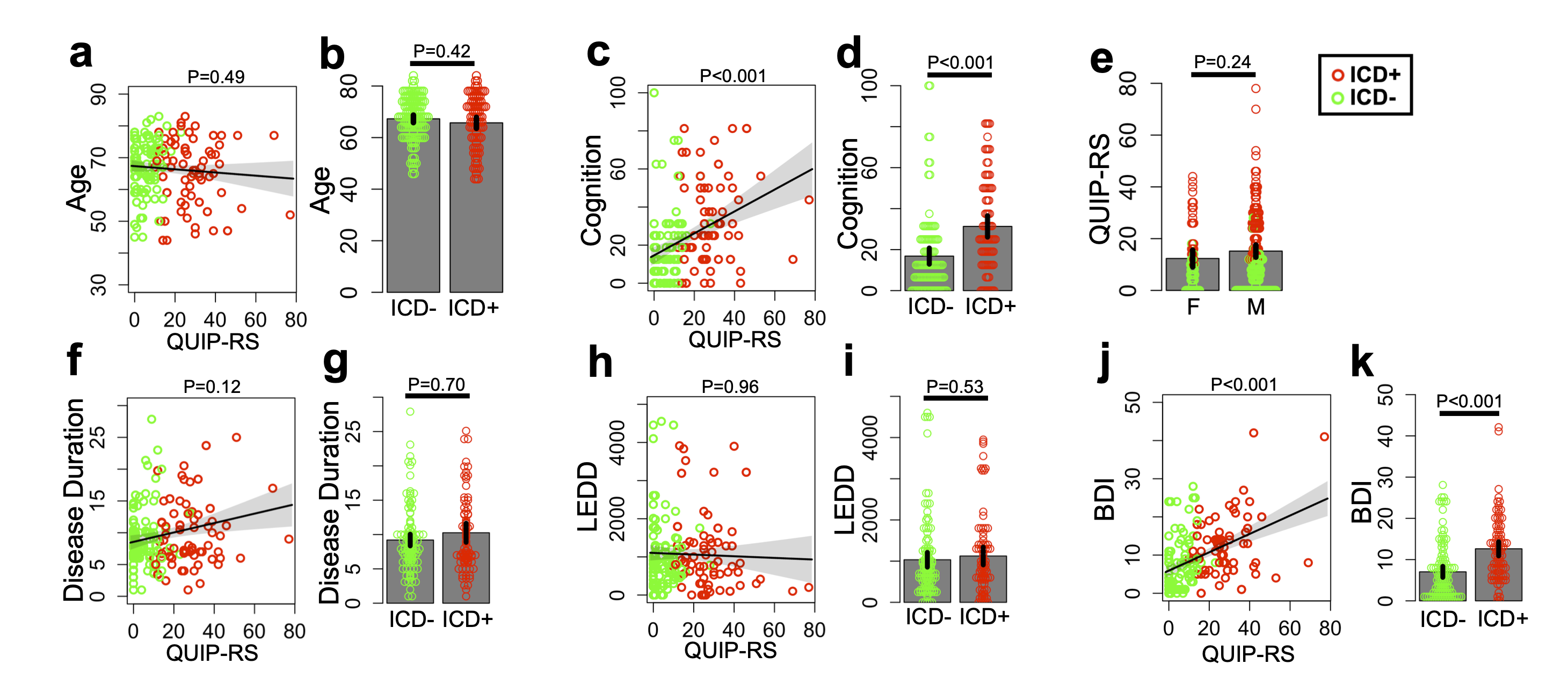

Supplement: Supplementary file 2 [file 41598_2020_69760_MOESM2_ESM.tiff]
